# Application of Mobile Signaling Data in Determining the Seismic Influence Field: A Case Study of the 2017 Mw 6.5 Jiuzhaigou Earthquake, China

**DOI:** 10.3390/ijerph191710697

**Published:** 2022-08-27

**Authors:** Xinxin Guo, Benyong Wei, Gaozhong Nie, Guiwu Su

**Affiliations:** 1 Institute of Geology, China Earthquake Administration, Beijing 100029, China; 2 Key Laboratory of Seismic and Volcanic Hazards, China Earthquake Administration, Beijing 100029, China

**Keywords:** mobile signaling, seismic influence field, rapid assessment, Jiuzhaigou earthquake

## Abstract

Seismic disasters are sudden and unpredictable, often causing massive damage, casualties and socioeconomic losses. Rapid and accurate determination of the scale and degree of destruction of the seismic influence field in an affected area can aid in timely emergency rescue work after an earthquake. In this study, the relationship between the changes in four types of mobile signaling data and the seismic influence field was explored in the 2017 Jiuzhaigou earthquake-hit area, China, by using the methods of comparative analysis, regression analysis and spatial autocorrelation analysis. The results revealed that after the earthquake, the number of mobile signaling significantly decreased. The higher the intensity, the more obvious the reduction of mobile signaling data and the later the recovery time. The Loginmac and WiFi data showed greater sensitivity than Gid and Station. There was a significant correlation between the changes in the mobile signaling numbers and the seismic intensity, which can more accurately reflect the approximate extent of the seismic influence field and the degree of actual damage. The changes in mobile signaling can provide a helpful reference for the rapid determination of seismic influence fields.

## 1. Introduction

As one of the most devastating natural disasters, earthquakes are sudden and unpredictable. The occurrence of seismic disasters causes not only extensive damage to buildings and social infrastructure but also many casualties [1,2]. Since the initial stage of an earthquake is often in the “information black box period”, the rapid determination of the seismic influence field represents a key and difficult issue for post-earthquake emergency rescue efforts [3]. With the progress of technical systems, some developed countries have established seismic influence field assessment systems [4,5,6,7,8,9]. China is one of the most earthquake-affected countries in the world. Faced with the challenges of its vast territory, complex topography and sparse seismic stations, many scholars have started to experiment with the assessment of seismic influence fields using techniques such as instrumental intensity, ShakeMap and remote sensing [10,11,12].

In recent years, the internet and spatial positioning technologies have developed. Social media platforms and geolocation-tagged information on the internet have become a new way to rapidly assess disasters and prepare post-disaster responses [13,14,15,16,17,18]. Many scholars are also trying to use user location information to assess the extent of seismic influence fields. For example, Shuai et al. [19] conducted spatial interpolation analysis to determine the extent of the seismic influence field based on user location information. Arapostathis et al. [20] used earthquake-related tweets with location information to create an isoseismal map on the southern coast of the Greek island of Lesvos. Taking the 2017 Jiuzhaigou earthquake as an example, Xing et al. [21] analyzed relevant spatiotemporal and semantic information from microblogs.

Unlike social media data, mobile signaling data can continuously track the spatial location, status and other information about mobile phone users through a base station with high time sensitivity and high accuracy [22,23,24,25,26,27]. Many scholars have applied mobile signaling data to study population mobility, transportation, land use and city planning [28,29,30,31,32,33,34]. The study of the seismic influence field based on mobile phone signaling data is also receiving increasing attention. For example, some scholars have used post-earthquake mobile signaling data to analyze the scale and trend of population movement after an earthquake [25,35,36]. Additionally, Moumni et al. [37] used mobile signaling data before and after an earthquake to analyze changes in citizens’ social responses to the earthquake. Li et al. [38] used mobile phone location data within 24 h after the Jiuzhaigou earthquake to conduct a time-by-time analysis and interpolated spatial simulations to estimate the deviation of the multidimensional population distribution. Pang et al. [39] compared the changes in mobile signaling data before and after an earthquake. Xia et al. [40] attempted to establish an intensity assessment model based on the rate of change in the number of mobile phone devices for eight earthquakes in China. Xing et al. [41] proposed a method for assessing the degree of disaster impact by combining social media data and mobile phone signaling data. Dai et al. [27] used a spatiotemporal clustering method to analyze post-earthquake population evacuations and determine earthquake impacts based on mobile signaling data. In combination with traditional empirical assessment models, the application of mobile signaling data can provide timely and powerful technical support for post-disaster emergency relief, thus further improving the efficiency of emergency relief [42]. However, the study of mobile phone signaling data is still in the exploration stage. The theoretical models and technical methods are still immature and need further empirical studies for improvement.

This study used the 2017 Mw 6.5 Jiuzhaigou earthquake in Sichuan Province, China, as an example and analyzed the changes in four types of mobile signaling data after the earthquake. This study explored the relationship between the changes in mobile signaling data and the local seismic influence field. We also attempted to establish an earthquake intensity assessment model and an interpolation analysis of the seismic influence field based on mobile signaling data to provide a reference for rapid assessment of the post-earthquake damage extent and the seismic influence field.

## 2. Methods and Data

### 2.1. Study Area and 2017 Jiuzhaigou Earthquake

**Figure 1:**
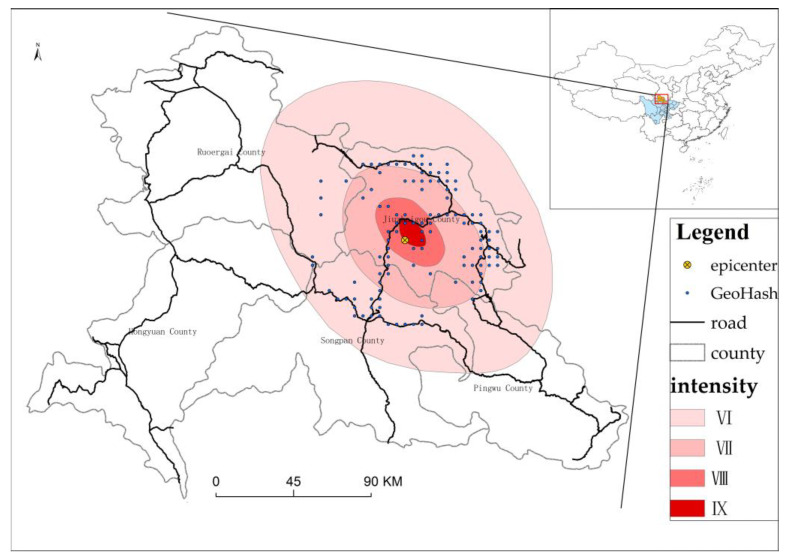
Location map of the study area.

The study area is the area impacted by the Mw 6.5 Jiuzhaigou earthquake, China (Figure 1). The Mw 6.5 Jiuzhaigou earthquake occurred at 21:19:46 on 8 August 2017, with the epicenter (33.20° N, 103.82° E) located in Jiuzhaigou County, Aba Prefecture, Sichuan Province, China. This area represents the intersection of the Huya Fault on the eastern boundary of the Bayan Har Block and the Tazang Fault in the eastern section of the East Kunlun Fault Zone. The maximum intensity of this earthquake was IX degrees, the long axis of isoseismal lines trended north–northwest and the total area with seismic intensities of VI degrees and above was 18,295 km^2^ [38]. This study area mainly covers areas with seismic intensities of VI degrees and above in Sichuan Province, China (Figure 1).

Jiuzhaigou County was the main area affected by the earthquake. Jiuzhaigou County is part of the Aba Tibetan and Qiang Autonomous Prefecture in northern Sichuan Province, China, and is bordered by Wenxian, Zhouqu and Diebe Counties in Gansu Province, China. The terrain is high in the west and low in the east, with an elevation of over 2000 m. The Jiuzhaigou Scenic Area is a well-known tourist attraction in China and abroad, and the county covers an area of 5290 km^2^ [43].

### 2.2. Methods

#### 2.2.1. Analysis of Mobile Signaling Changes

We selected mobile signaling data from 20:00 on 7 August to 0:19 on 8 August (referred to below as the day before the earthquake) and from 20:00 on 8 August to 0:19 on 9 August (referred to below as the day of the earthquake). To quantitatively evaluate the change in mobile signaling data before and after the earthquake, this paper defines a decommission rate *R* index to measure it. The decommission rate *R* is the rate of change in the number of devices *X* at time *t* in the GeoHash square *g* on the day of the earthquake for mobile signaling *i* relative to the number of devices *X*′ at the same time *t* on the day before the earthquake. It can be calculated with the following formula:

*R_gti_* = [(*X*′*_gti_* − *X_gti_*)/*X*′*_gti_*] × 100% (1)

where *R_gti_* is the decommission rate of mobile signaling *i* at time *t* in GeoHash grid *g*; *X*′*_gti_* is the total number of devices at time *t* in GeoHash grid *g* on the day before the earthquake for mobile signaling signal *i;* and *X_gti_* is the total number of devices at time *t* in GeoHash grid *g* on the day of the earthquake for mobile signaling *i*.

The decommission rate reflects the magnitude of the increase or decrease in the number of devices counted by mobile signaling, which characterizes the degree of response of mobile signaling to the earthquake shock. If the decommission rate is a positive value, the number of devices counted after the earthquake is reduced; the greater the value is, the greater the reduction in mobile phone signaling after the earthquake. If it is negative, the number of devices counted after the earthquake increases; the greater the absolute value is, the greater the increase in the number of mobile phone signals after the earthquake. If the decommission rate tends to 0, the number of devices counted after the earthquake does not change significantly compared with that at the same time before the earthquake.

Furthermore, through the spatial overlay analysis of mobile signaling data and intensity zones, we also analyzed the change in mobile signal data in different intensity areas. The decommission rates of the intensity zones are calculated by an arithmetic average method, i.e., *R_gni_* (the n-minute average rate of decommission of mobile phone signaling *i* in GeoHash square *g*). The specific equation is as follows:

(2) $$R_{\mathrm{gni}}=\sum_{t}^{t+n}R_{igt}/n$$

Additionally, to reduce the influence of random fluctuations on the change in the decommission rate over time, this study also processed the time-varying series of mobile phone decommission rates by the moving average technique to eliminate the influence of chance variation factors. The specific processing equation is as follows:

*F_git_* = (*R_git_* + *R_git*+1*_* + *R_git*+2*_* + … + *R_git*+*N_*)/*N* (3)

where *F_git_* is the moving average of mobile signaling *i* at *N* minutes after the start of moment *t* in GeoHash grid *g*; *R_git_* is the decommission rate of mobile signaling *i* at moment *t* in GeoHash grid *g*; *R_git_*_+1_ and *R_git_*_+2_ to *R_git_*_+*N*_ are the decommission rates of mobile signaling *i* at 1 min and 2 min to *N* minutes after moment *t*, respectively.

#### 2.2.2. Analysis of the Relationship between Mobile Signaling Data Changes and Seismic Intensities

The mobile signaling data may change significantly due to the seismic impact. This variation also shows variability with the region of the affected area, which provides the possibility of using mobile phone location data to determine the extent of the seismic influence field. We used Pearson correlation analysis and a multiple linear regression model to study the relationship between the variation in mobile signaling data and seismic intensities. With SPSS software (IBM, Armonk, NY, USA), we conducted a correlation and regression analysis of the mobile signaling data with the seismic intensities. By comparing and analyzing multiple models, we selected the model with the best fitting effect as the judgment model for the seismic influence field in the study area.

#### 2.2.3. Kriging Interpolation Methods

Spatial interpolation analysis is a geostatistical method that uses information from sampled points to predict information about unknown regions. This study conducted interpolation analysis based on the decommission rate *R*, which varied spatially according to the GeoHash grid and has spatial correlation. Kriging is the method of interpolation deriving from the regionalized variable theory. It can represent the spatial variation of attributes and is more accurate and realistic than other methods [39,44]. Therefore, this study used kriging interpolation to carry out a spatial interpolation analysis of the decommission rate *R*. In addition, we overlaid the analysis with the published intensity map to further validate the analysis with the interpolation results.

### 2.3. Data

#### 2.3.1. Data Acquisition

The mobile signaling data in this study come from a third-party information push company. The mobile phone signaling data are in GeoHash format with 5-bit precision. GeoHash encoding is an address encoding method that can encode two-dimensional spatial latitude and longitude data into a string. The longer the string is, the higher the accuracy of the representation. GeoHash coding is used for spatial retrieval, i.e., the entire map plane is divided into several rectangular squares, and each square is coded. The 5-bit GeoHash represents a rectangular area with dimensions of 4.9 km × 4.9 km. The data in this study are based on location acquisition, but they have been anonymized and do not include any personal privacy information.

This study cited the fatalities distribution points and secondary hazard potential points in the Mw 6.5 Jiuzhaigou earthquake to further validate the interpolation results. These two data were obtained from the Mw 6.5 Jiuzhaigou earthquake post-disaster assessment report and the Chinese National Natural Resources and Geospatial Basic Information Database [45].

#### 2.3.2. Mobile Signaling

Through preliminary research, four types of mobile signaling data—Gid, Station, Loginmac and WiFi—were selected in this paper. Gid is a count of the number of mobile devices in each GeoHash square in the area range per minute based on multiple location methods to obtain mobile device location data for the service. The Station data represent the number of stations based on the information of scanned base stations reported by mobile devices at regular intervals, and the number of stations per unit time in each GeoHash grid is counted. Loginmac is the number of Loginmac per unit time in each GeoHash grid of the region based on the network connection status reported by the connected mobile devices at regular intervals. WiFi is a count of the number of WiFi hotspots per unit time in each GeoHash grid of the region based on the scanned WiFi hotspot information reported by mobile devices at regular intervals. If there is a drastic change in the number of devices in a GeoHash unit within a time slice, there may be large-scale anomalies in the range and time period, where device data are not properly reported or the number of devices has skyrocketed, thus suggesting that an earthquake or other sudden disaster has occurred in the region [39].

#### 2.3.3. Data Overview

The data used in this study were a 5-bit GeoHash grid with data reported on the day before the earthquake (7 August, 20:00–8 August, 0:19) and the day of the earthquake (8 August, 20:00–9 August, 0:19). We collected the data from the area within 50 km of the epicenter (33.20° N, 103.82° E), mainly covering the area of VI degrees and above in Sichuan Province, China (Figure 1). The Gid, Station, Loginmac and WiFi numbers were 112, 110, 91 and 102, respectively. Table 1 shows the total number of devices counted and the decommission rates on the days before and after the earthquake for the four types of signaling data. On the day before the earthquake, the number of devices collected by Gid, Station, WiFi and Loginmac reached 93,482, 65,056, 597,533 and 106,275, respectively. However, on the day of the earthquake, the number of devices collected from Gid, Station, WiFi and Loginmac dropped to 67,292, 48,405, 321,410 and 46,299, respectively. The decommission rate of WiFi was largest, followed by Loginmac, indicating that WiFi and Loginmac are more sensitive to the impact of local earthquake hazards.

Regarding the signaling data reported in different intensity areas (Table 2), Gid had 59, 36, 13 and 4 GeoHash grids in the VI-, VII-, VIII- and IX-intensity zones, respectively. Station had 33, 31, 13 and 4 GeoHash grids in the VI-, VII-, VIII- and IX-intensity zones, respectively. WiFi had 53, 35, and 4 GeoHash grids in the VI-, VII-, VIII- and IX-intensity zones, respectively. Finally, Loginmac had 51, 26, 10 and 4 GeoHash grids in the VI-, VII-, VIII- and IX-intensity zones, respectively.

## 3. Results

### 3.1. Analysis of the Overall Change in Regional Mobile Signaling

#### 3.1.1. Daily Variation in the Number of Devices on the Day before the Earthquake

Influenced by people’s work and rest habits, the number of devices generating mobile signaling varied among different periods. We conducted a minute-by-minute analysis of the changes in the four types of signaling data from 20:00 on 7 August to 0:19 on 8 August, the day before the Jiuzhaigou earthquake, to obtain the daily variation in the four types of signaling data in the absence of seismic activity.

**Figure 2:**
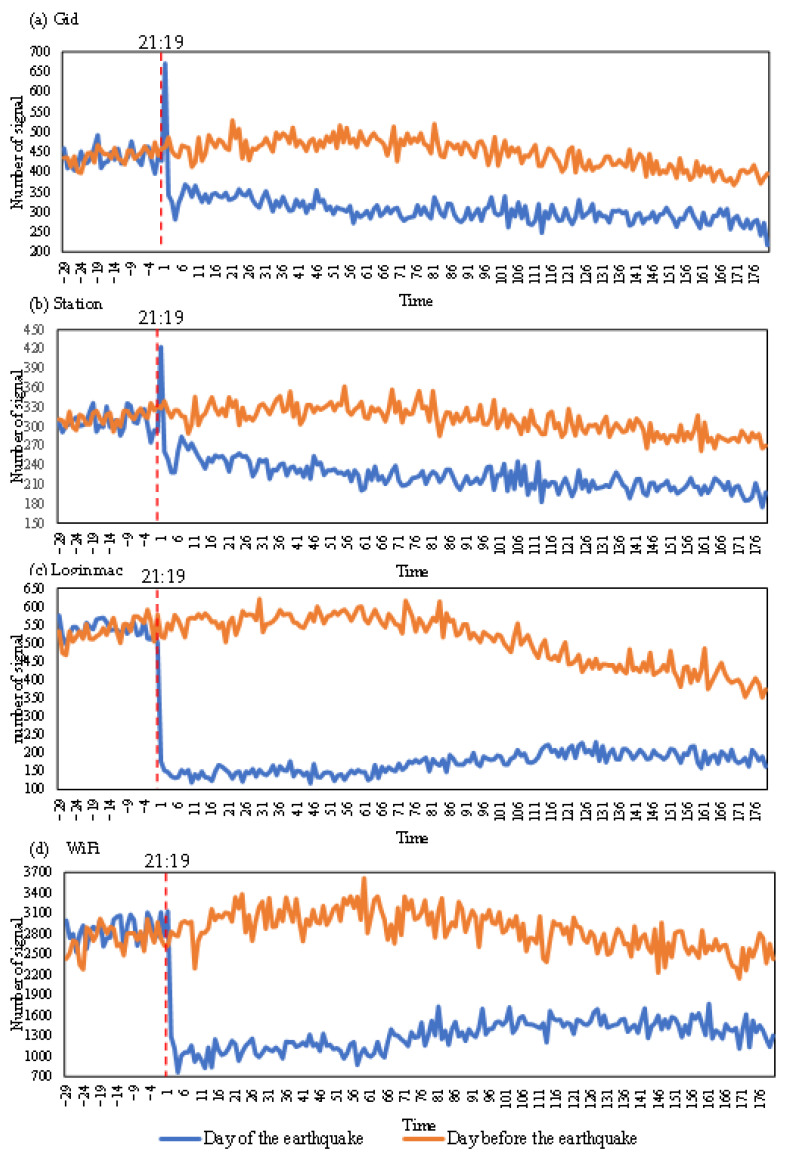
The minute-by-minute changes in the number of devices associated with the four types of signaling data. On the horizontal axis, 1 represents 1 min after 21:19, i.e., 21:20; 2 represents 2 min after 21:19, i.e., 21:21, …, and 180 represents 0:19 the next day. −1 represents 1 min before 21:19, i.e., 21:18, 2 represents 2 min before 21:19, i.e., 21:17, …, and −29 represents 20:50.

Figure 2 shows the minute-by-minute variation in the number of devices associated with the four types of mobile signaling data, and these observations can be divided into three phases for the day before the earthquake. The first stage was from 20:50 to 21:40, when the number of devices generally increased. From 21:40 to 22:40 was the second stage, when the number of devices was the highest during the whole study period, and the overall number of devices fluctuated slightly in the high range. The third phase was from 22:40 to 0:19, which showed a fluctuating downward trend in the number of devices. The occurrence of these changes is mainly related to the resting habits of residents. In the first stage, people gradually start to return home or conduct rest activities after finishing their work. Thus, the number of devices in this stage showed a fluctuating increase. In the second stage, people are generally in a state of leisure and recreation but not sleeping, so the number of collected devices is relatively high. In the third stage, people started to sleep, the number of people using the devices gradually decreased and the decline rate gradually increased over time. Among the four types of signaling data, WiFi signaling was associated with the largest number of devices. Station signaling was associated with the least number of devices.

In addition, the maximum number of devices occurred at different times for the four types of signaling data, which indicated that the different types of signaling data responded at times to changes in people’s work and rest habits. Loginmac exhibited the largest change in the study period, which indicated that Loginmac is the most sensitive to changes in people’s activities.

#### 3.1.2. Signaling Changes on the Day of the Earthquake

The time of the Jiuzhaigou earthquake was 21:19 on 8 August 2017. From the minute-by-minute changes in the number of devices in Figure 2 and the sudden changes in the four types of signaling data in Table 3, we found that all four types of signaling data exhibited sudden changes within 3 min after the earthquake.

The sudden change in Gid occurred at 21:20, and the number of devices varied steadily between 250 and 350 from 20:50 to 21:19. At 21:20, there was a sudden increase, reaching a maximum value of 671 during the acquisition period. Then, a sudden decrease occurred at 21:21, dropping to 342. At 21:23, the number of devices dropped to 281 after three consecutive minutes of decline. Then, it exhibited slight fluctuations up and down, with an overall downward trend.

The sudden change in Station occurred at 21:20, and the number of devices varied steadily between 400 and 500 from 20:50 to 21:19. At 21:20, there was a sudden increase, reaching a maximum value of 423. At 21:21, there was a sudden decrease, down to 261. Then, the number of devices maintained a slight up-and-down fluctuation with an overall decreasing trend.

The sudden change in Loginmac occurred at 21:20, and the number of counted devices varied steadily between 500 and 600 from 20:50 to 21:19. The number decreased abruptly to 176 at 21:20 and then exhibited small fluctuations with an overall upwards trend.

The sudden change in WiFi occurred at 21:21, and the number of devices varied steadily between 2500 and 3500 from 20:50 to 21:20. The number of devices suddenly decreased at 21:21, dropping to 1287, and then exhibited small fluctuations with an overall upward trend.

The above analysis showed that the number of devices associated with the four types of signals experienced sudden changes after the earthquake. Two abrupt change points occurred for Gid and Station. The trends of Gid and Station were similar, but the change in the Gid data was larger than that in the Station data. The magnitudes of change in Loginmac and WiFi were similar. However, the sudden decrease in Loginmac occurred at 21:20, and the sudden change lasted 5 min. The sudden change in WiFi occurred at 21:21 and lasted 3 min.

#### 3.1.3. Signaling Changes between the Day of and the Day before the Earthquake

Figure 2 shows the change in the number of devices before and after the earthquake. Before the mutation point, the number of devices on the day of the earthquake was comparable to that on the day before the earthquake, consistent with the habits of the local people. The number of devices counted on the day of the earthquake after the abrupt change point was much lower than that on the day before the earthquake. The change in Station signals was the smallest, while the change in WiFi was the largest. The number of devices associated with the four types of signaling data changed significantly after the earthquake, and the number of devices associated with Loginmac varied the most. Gid and Station showed decreasing trends after the abrupt change point, while WiFi and Loginmac showed increasing trends after 23:00.

#### 3.1.4. Analysis of the Decommission Rate for Mobile Signaling

**Figure 3:**
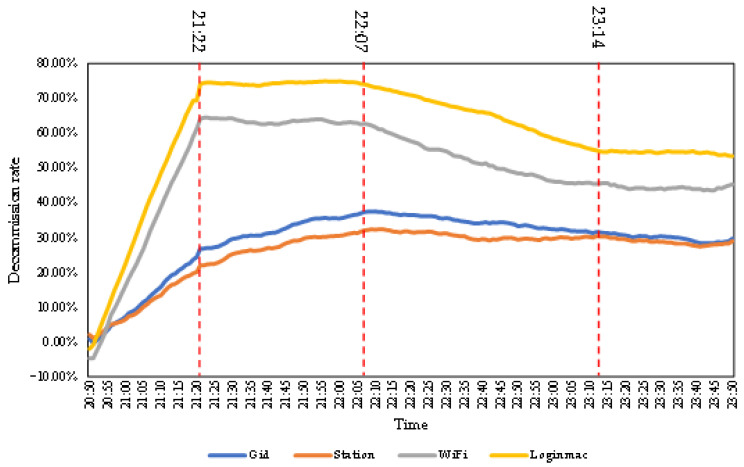
The 30 min moving average change in decommission rate for the four types of mobile signaling.

Figure 3 shows the 30-min moving average change in the decommission rate of the four types of mobile signaling data. The Gid and Station data can be divided into three phases. The WiFi and Loginmac data can be divided into four phases. The first phase of all four types of signaling was the surge phase. During the 30-min period starting from 20:50, when the earthquake had not yet occurred, the decommission rate for all four types of signaling was close to zero. At 21:19, when the earthquake occurred, the number of devices associated with the four types of signaling changed abruptly, and the corresponding decommissions rate also changed sharply.

The second phase was from 21:22 to 22:07, when the WiFi and Loginmac data had slight and stable changes. The range of the decommission rate was 60–70% for WiFi, while that of Loginmac was 70–80%. Loginmac was more sensitive to the earthquake in this phase. The Gid and Station data in this phase exhibited a slow rise. The number of devices was increasing before the earthquake, while the number of devices was still low on the day of the earthquake. Thus, the decommission rate of the phase was still in the rising stage. Compared with the first stage, some mobile signals recovered, so the rise was flatter in this stage.

The third phase in the Gid and Station data lasted from 22:07 to 23:50, during which these signals showed small fluctuations and stable changes. Gid showed a slight downward trend from 22:07 to 23:14. The decommission rates of Gid and Station varied steadily at the same level from 23:14 to 23:50. The third phase of WiFi and Loginmac was from 22:07 to 23:14. The decommission rates showed a slight decline.

In addition, WiFi and Loginmac had a fourth phase from 23:14 to 23:50, during which they were relatively stable.

### 3.2. Change in Mobile Signaling in Different Intensity Areas

There are differences in mobile signaling among different seismic intensity areas. By analyzing the changes in mobile signaling data before and after the earthquake, we can find the relationship between seismic intensity and mobile signaling data, which can help to judge the scale and damage degree of the seismic influence field.

#### 3.2.1. Signaling Changes in Various Intensity Areas

Table 4 shows the number of devices in different intensity areas for the four types of signaling data. On the day before the earthquake, 44,206 Gid devices, 33,334 Station devices, 64,554 Loginmac devices and 235,963 WiFi devices were counted in the area that had an intensity of VI. In the VII-intensity area, the numbers of devices associated with Gid, Station, Loginmac and WiFi were 7688, 5509, 7953 and 23,431, respectively. A total of 22,346 Gid devices, 14,290 Station devices, 17,564 Loginmac devices and 198,243 WiFi devices were counted in the VII-intensity area. In the IX-intensity area, the numbers of devices associated with Gid, Station, Loginmac and WiFi were 19,242, 11,923, 16,204 and 139,896, respectively. On the day of the earthquake, 39,222 Gid devices, 29,177 Station devices, 37,999 Loginmac devices and 191,027 WiFi devices were counted in the VI-intensity area. In the VII-intensity area, the numbers of devices associated with Gid, Station, Loginmac and WiFi were 5504, 4301, 1704 and 9335, respectively. A total of 13,177 Gid devices, 8799 Station devices, 4151 Loginmac devices and 77,016 WiFi devices were counted in the VII-intensity area. In the IX-intensity area, the numbers of devices associated with Gid, Station, Loginmac and WiFi were 9389, 6128, 2445 and 44,032, respectively. Overall, the number of devices on the day of the earthquake was lower.

Loginmac had the highest decommission rate in all four intensity zones. Except in the VI-intensity area, Station had the lowest decommission rate, and Gid had the lowest decommission rate in the VI-intensity area. In different intensity zones, the decommission rate gradually increased as the intensity increased. A steep rise occurred from VI-intensity to VII-intensity. The above analysis showed that Loginmac was more sensitive to the earthquake, and the number of devices varied greatly.

#### 3.2.2. Analysis of the Minute-by-Minute Change in the Number of Devices in Different Intensity Areas

**Figure 4:**
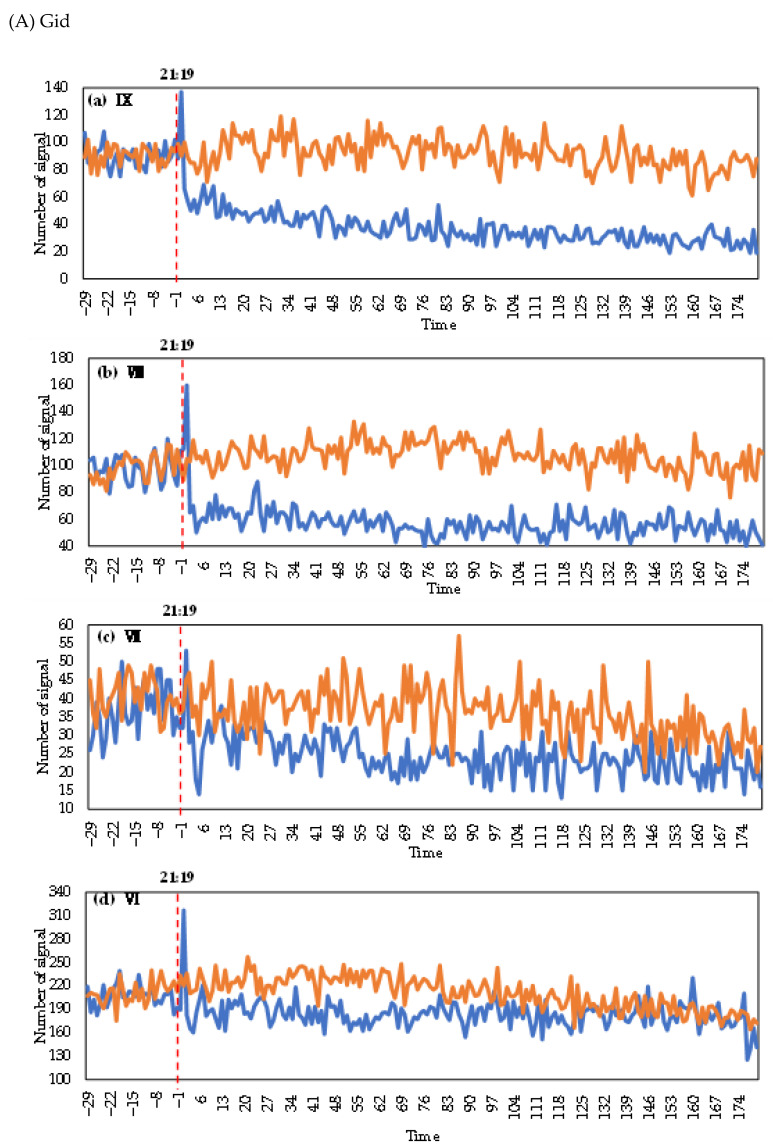

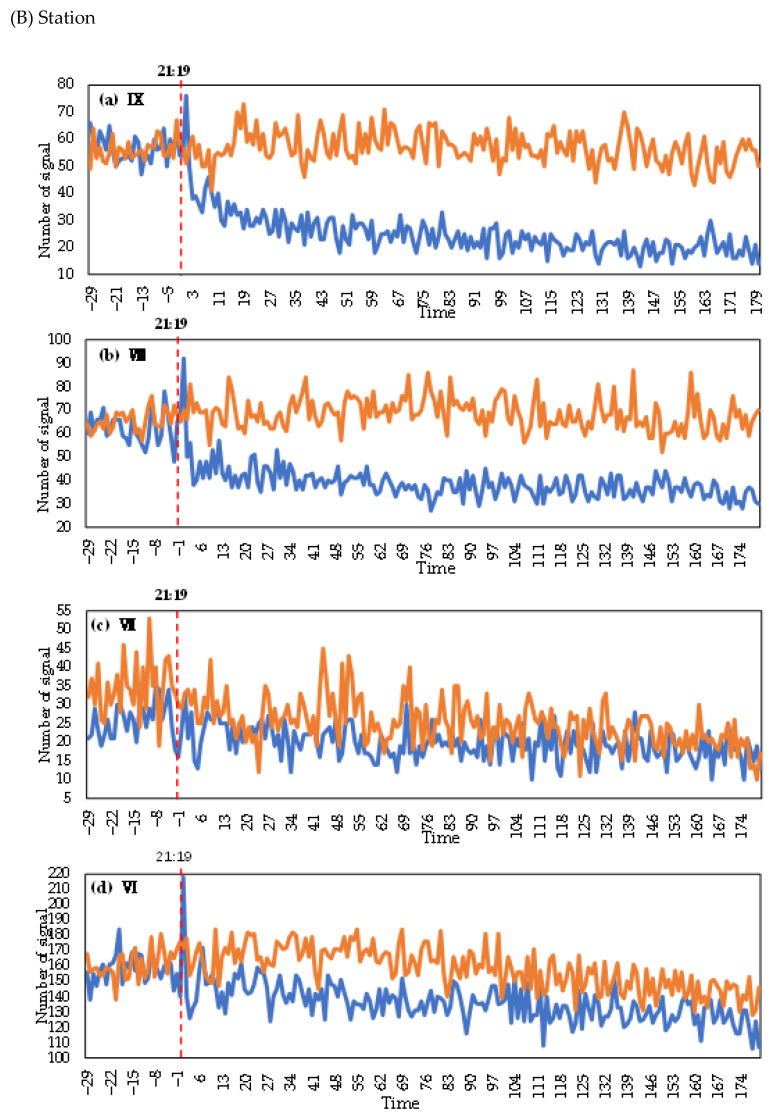

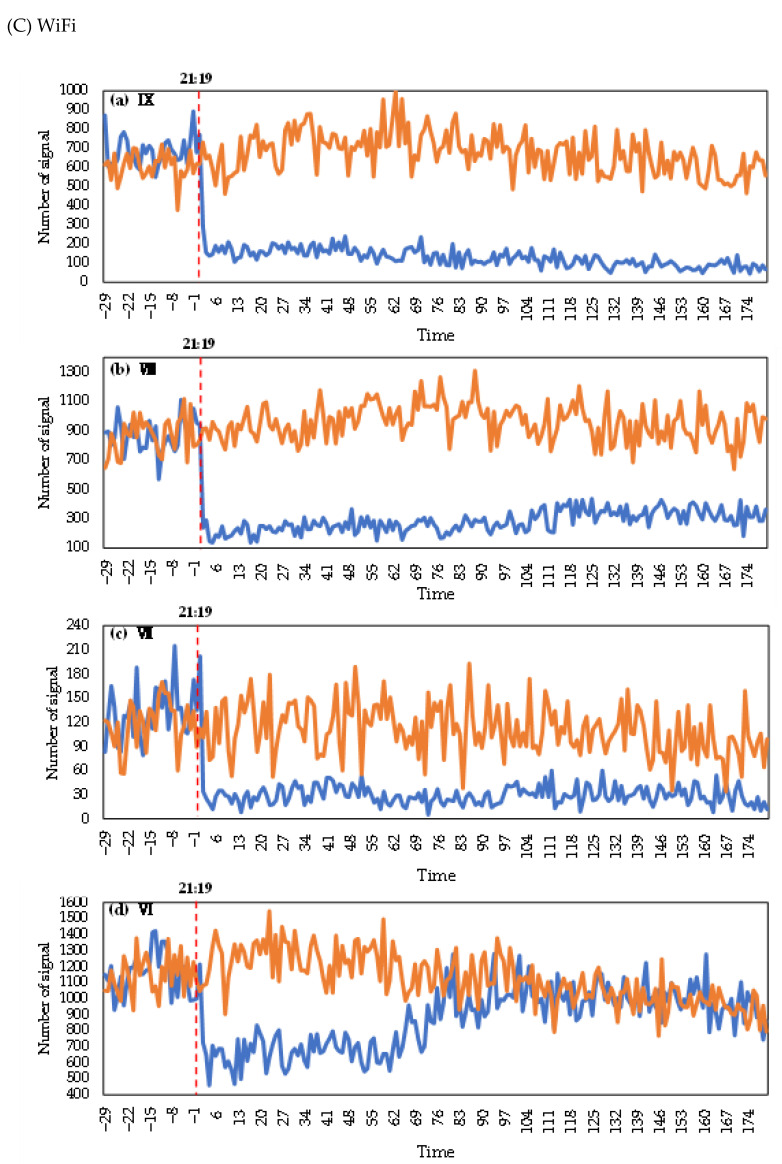

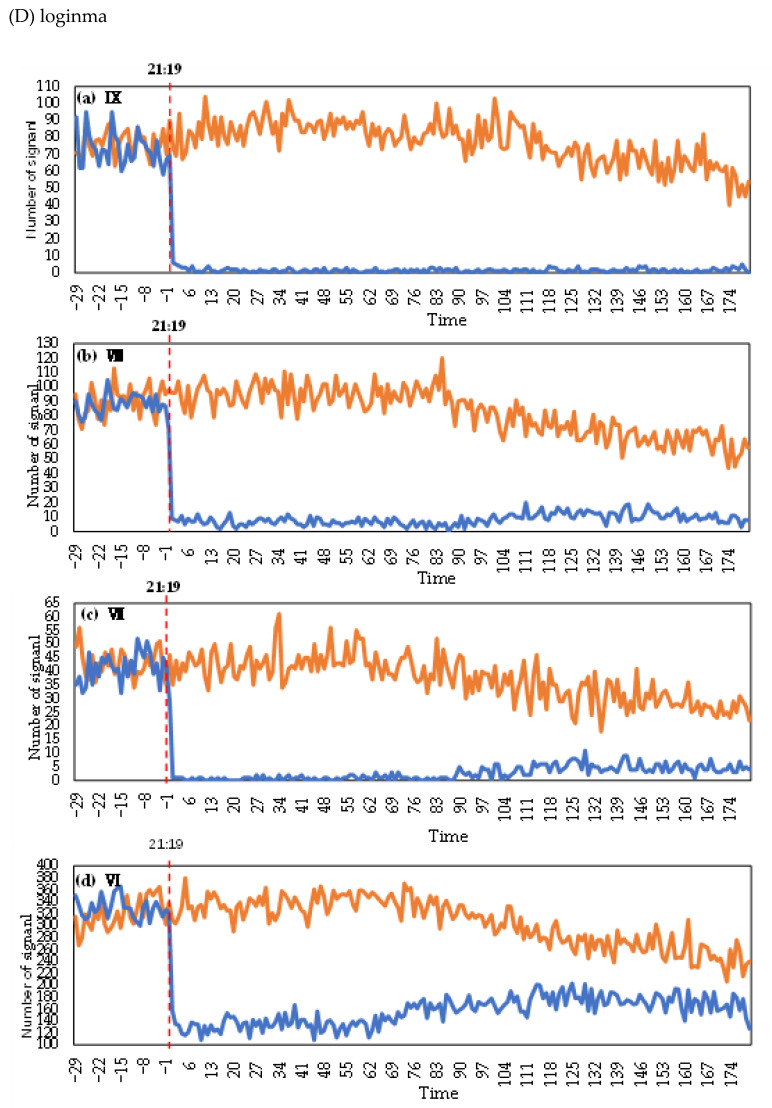
The change in the number of devices associated with the four types of mobile signaling minute-by-minute in different intensity zones. On the horizontal axis, 1 represents 1 min after 21:19, i.e., 21:20; 2 represents 2 min after 21:19, i.e., 21:21, …, and 180 represents 0:19 the next day. −1 represents 1 min before 21:19, i.e., 21:18, 2 represents 2 min before 21:19, i.e., 21:17, …, and −29 represents 20:50.

Figure 4 shows the minute-by-minute variation in the number of devices associated with the four types of signaling data (Gid, Station, Loginmac and WiFi) in different intensity zones.

From Figure 4A, the trends of Gid in different intensity areas were the same as the overall trends. The comparison of the different intensity areas showed that the intensity magnitude and the number of devices after the earthquake exhibited positive trends. The IX-intensity zone exhibited the largest changes, while the VI-intensity zone exhibited the smallest changes.

Figure 4B shows the changes in the number of Station devices in different intensity areas. After the earthquake, the number of devices in the IX-intensity zone varied the most from the day before the earthquake, followed by that in the VIII-intensity zone. In the VII-intensity zone, the number of devices after the earthquake changed only slightly. The reason may be that the area included in the VII-intensity zone is the most mountainous and is sparsely populated, so the base number of Station devices is small. After an earthquake, the change in the number of devices increases significantly with increasing seismic intensity.

Figure 4C shows the change in the number of WiFi devices in different intensity zones. After the earthquake, the higher the seismic intensity is, the larger the decrease in the number of devices. WiFi recovered faster in the VI-intensity zone than in other zones due to less seismic damage. One hour after the earthquake, the number of devices started to show an upwards trend and returned to the usual daily pattern by 23:00.

Figure 4D shows the change in the number of Loginmac devices in different intensity areas. After the earthquake, the number of devices in all four intensity areas decreased significantly, and the decrease was positive with the intensity. In addition, after the earthquake, the number of devices rebounded earlier in the lower-intensity areas. The zones with intensities of VIII, VII and VI showed slight upwards trends after 23:00, 22:50 and 22:20, respectively. The greater the impact of the earthquake in high-intensity areas, the longer it took for the infrastructure to recover, whereas the infrastructure recovered quickly in the less-damaged, low-intensity areas. Therefore, after the earthquake, Loginmac in lower-intensity areas recovered earlier.

The change trends of the four types of signaling data in different intensity zones were consistent with the overall change trends. Compared with the pattern on the day before the earthquake, the change in the number of devices was positively correlated with the intensity after the earthquake, except for the Station data in the VI-intensity zone, which exhibited slight changes. Both Loginmac and WiFi decreased greatly after the earthquake. The number of devices started to recover in low-intensity zones. Loginmac showed varying degrees of recovery in the VIII-, VII- and VI-intensity zones, while WiFi appeared to recover only in the VI-intensity zone.

#### 3.2.3. Thirty-Minute Moving Average Change in the Decommission Rate for Different Intensity Areas

**Figure 5:**
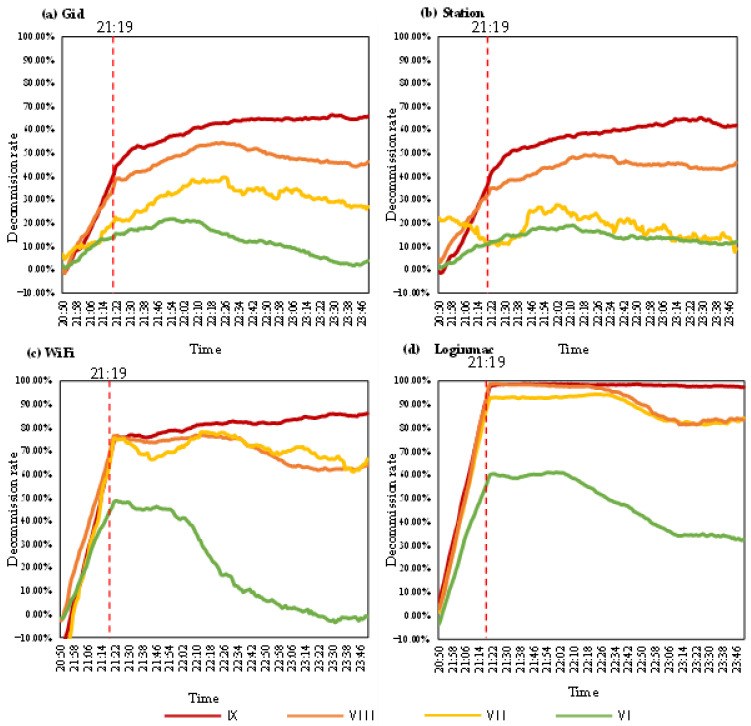
The 30-min moving average variation of decommission rate in different intensity zones for the four types of mobile signaling.

Figure 5 shows the changes in the 30 min moving average of the decommission rate of the four types of signaling data in different intensity areas. The trends in different intensity zones were the same as their overall trends. The greater the intensity was, the greater the corresponding decommission rate and the magnitude of change.

In the IX-intensity area, the decommission rate and the change amplitude of all four types of signaling data were the highest and could be roughly divided into two phases. The first phase was the sudden change phase, which mainly occurred during the period from 20:50 to 21:19. The earthquake occurred at 21:19, and the four types of signaling changed abnormally in response to the earthquake. The second phase was the slight increasing phase from 21:19 to 23:50. Although the sudden change ended, there was heavy damage to the infrastructure in the high-intensity zones due to the severe impact of the earthquake. There was still the risk of aftershocks, and the residents in the IX-intensity zone may have continued to evacuate the area during this time, so the decommission rate was still in the rising phase. In addition, the decommission rate values and variations in WiFi and Loginmac were higher than those of Gid and Station. The decommission rate of Loginmac tended to 1, which indicated that Loginmac might be more sensitive to earthquakes in high-intensity zones.

In the VIII-intensity region, the change trends of the four types of signaling data were similar to the overall change trends. The Gid and Station data could be divided into three phases: the first phase was a sudden change phase, the second phase was a slight increase phase, and the third phase was a small decrease phase. Gid showed a more significant decline in the third stage, while Station was more stable. The WiFi and Loginmac data could be divided into four phases: the first phase was an abrupt change phase, the second phase was a stable change phase with a high decommission rate, the third phase was a slight decline phase and the fourth phase was a stable change phase with a low decommission rate. Compared with Gid and Station, WiFi and Loginmac had a significant recovery phase, which indicated that WiFi and Loginmac were more capable of post-earthquake signaling recovery.

In the VII-intensity area, the trend of the decommission rate for Gid was similar to the first two intensity zones, but the value and the magnitude of the change decreased considerably. The Station data fluctuated more in the VII-intensity area. The first stage was from 20:50 to 21:31, showing fluctuating changes above and below 0.1. A sudden increase occurred in the second phase (21:31 to 21:44), but the increase was smaller than that in the IX- and VIII-intensity zones. The third phase, from 21:44 to 23:50, showed a fluctuating decline. Both WiFi and Loginmac showed sudden and significant increases in the first stage. WiFi showed a decline followed by an increase in the second stage and a fluctuating decline in the third stage. Loginmac showed a similar trend to that in the VIII-intensity zone in the second, third and fourth stages.

In the area with intensity VI, the values and variations in the four signaling decommission rates were smaller due to the lesser impact of the earthquake. The Gid and Station data in this intensity zone can be divided into two phases. The first phase was the rising phase. Because this zone was affected by the earthquake, the magnitude of the abrupt change was smaller, and the data exhibited only a gentle upwards trend. The second phase was the decreasing phase, where the signaling gradually recovered and gradually returned to pre-earthquake levels. Consequently, the decommission rate tended to 0 in this phase. The WiFi and Loginmac data in this intensity zone could still be divided into four phases. The values and the magnitude of change were lower than those in the high-intensity zones.

### 3.3. The Relationship between the Change in Mobile Signaling and Intensity

We selected the average 5-min post-earthquake decommission rate of the four types of signaling (Gid, Station, WiFi and Loginmac) to study the relationship between the change in mobile signaling and intensity. Gid and Station exhibited changes at 21:20. To reduce the impact of these abrupt changes, the data series of these two signals for the 5 min after the earthquake did not contain data at 21:20.

#### 3.3.1. Correlation Analysis

Table 5 shows the correlation between the intensity of mobile signaling *i* (1 is Gid; 2 is Station; 3 is WiFi; 4 is Loginmac) in GeoHash grid *g* and the epicentral distance, as well as the 5-min post-earthquake average decommission rate. *I_gi_* is the intensity of mobile signaling *i* in GeoHash grid *g*. *D_gi_* is the epicentral distance of mobile signal *i* in GeoHash grid *g*. *R_g5i_* is the 5-min post-earthquake average decommission rate of mobile signaling *i* in GeoHash grid *g*.

As shown in Table 5, the correlation between the epicentral distance of all four types of mobile signaling data and their intensities was significant at the 0.01 confidence level. The decommission rates of Gid and Station were significantly correlated with the intensity at the 0.05 confidence level, while WiFi and Loginmac were significantly correlated at the 0.01 confidence level. The intensity of all four types of mobile signaling data was negatively correlated with the epicentral distance, and the intensity of WiFi was the most highly correlated with the epicentral distance. The intensity of the four types of signaling was positively correlated with the decommission rate. The correlation between the intensity and the epicentral distance was greater than the correlation between the intensity and the decommission rate.

#### 3.3.2. Regression Analysis

We removed the noise in the four types of signaling data. Two-thirds of the data were randomly selected as test data with the epicentral distance to build an intensity assessment model, and one-third of the data were randomly selected as validation data. The test data of Gid, Station, Loginmac, and WiFi were 50, 44, 44, and 35, respectively. The validation data of Gid, Station, Loginmac, and WiFi were 25, 22, 22, and 17, respectively.

Table 6 shows the seismic intensity assessment models based on the average decommission rate and epicentral distance of the four types of signaling data. All four models were significant at the 95% confidence level. In terms of the model fitting effect, the model built based on Gid had the highest R^2^ value (0.822) and the best fitting effect. In terms of model validation, the model built based on WiFi had the highest accuracy of 86%.

### 3.4. Spatial Interpolation Analysis of Mobile Signaling

We removed the noise in the four types of signaling data, carried out interpolation analysis on these four types of data based on the 5 min post-earthquake decommission rate and overlaid the interpolation results with the Jiuzhaigou intensity map. The number of sample points for Gid, Station, WiFi and Loginmac data was 75, 66, 66 and 52, respectively.

**Figure 6:**
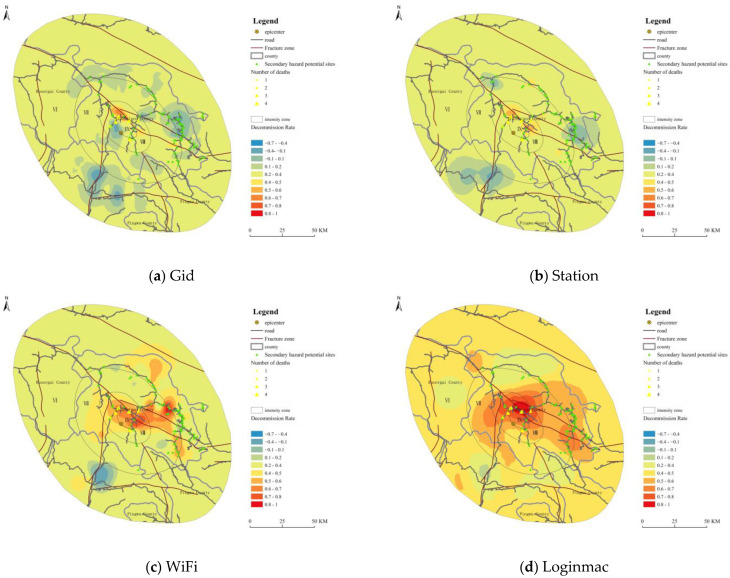
Interpolation results of the decommission rate.

Figure 6a shows the interpolation results from the Gid data, with the higher decommission rate values mainly concentrated in the area north of the epicenter above the VIII-intensity zone. In the interpolated results of the Gid data, the trends of the decommission rate and meizoseismal areas were not obvious. The interpolation results were relatively poor. Figure 6b shows the interpolation result for Station data, with two meizoseismal areas forming to the north and east of the epicenter, both of which were located at road crossings and had fatalities distributed in their vicinity. Compared to Gid data, the interpolation results based on the decommission rate of the Station data were better. However, the delineation of the meizoseismal area was not obvious, and the interpolation results were still unsatisfactory. Figure 6c shows the WiFi interpolation result, which is relatively good compared to that of the Gid and Station data. The decommission rate indicated a more pronounced range of extreme seismic zones distributed to the east of the epicenter and extending to the east, where all the fatalities of this earthquake were located. Figure 6d shows the interpolation result of the Loginmac data. Compared to the first three signaling types, the interpolation results of the Loginmac data had clear zones, and the meizoseismal area was extensive. The decommission rate in areas with intensities greater than VIII was mainly higher than 0.5, and the fatalities were all in areas where the decommission rate was higher than 0.5.

The overall fit results for the four types of signaling data were consistent with the published intensity map, and the high decommission rate areas were distributed within the high-intensity zones. The direction of change of the decommission rate was consistent with the road extension direction, and the distribution points of the fatalities located in the high decommission rate areas. This indicated that all four types of signaling data could provide a helpful reference for delineating the seismic influence field. Among them, the Lognimac data had the best interpolation, which could indicate the extent of the meizoseismal area and the direction of the meizoseismal area and the direction of change and provide helpful information for rapid emergency rescue.

## 4. Discussion

### 4.1. Response of Mobile Signaling to Earthquake Effects

Mobile signaling data could exhibit abnormal changes when affected by an earthquake. Our results found that the four types of mobile signaling experienced a cliff-like drop within 5 min after an earthquake. A quantitative metric, the decommission rate, was defined to characterize the degree of impact by a seismic event. Among the four signaling types, Loginmac had the highest decommission rate, followed by WiFi, while Station had the lowest decommission rate. Loginmac and WiFi showed a clear and significant decline phase, while Gid and Station showed a later and slight decline phase. The magnitude of change was also larger for Loginmac and WiFi. Therefore, in terms of earthquake sensitivity and post-earthquake recovery response, Loginmac and WiFi are more responsive than the other signaling types. We also found that the changes in mobile signaling were consistent with the changes in residents’ work and rest habits when not affected by an earthquake or other unexpected events. In conclusion, by comparing the changes in mobile signaling before and after the earthquake, it may be possible for us to determine the seismic impact.

### 4.2. Mobile Signaling Changes in Response to the Degree of Disruption

The intensity is a reflection of the degree of earthquake damage. We analyzed the situation of the four types of mobile signaling in different intensity areas. The results showed that the variation in the number of devices was more significant at higher intensities. By comparing the variations in signaling data in different intensity zones, we found that none of the four types of signaling recovered in the intensity IX zone. In the other three intensity areas, the lower the intensity was, the greater the recovery magnitude was, and the earlier the recovery time occurred. The decommission rate in different intensity areas showed that the greater the intensity, the greater the decommission rate. The decommission rate of all four types of signaling did not show a dropout phase in the IX intensity zone. In the other three intensity areas, the lower the intensity was, the earlier the dropout phase time occurred. The variation in mobile signaling data in different intensity zones in this study is consistent with the various patterns of mobile signaling data in different damage classification zones in Xing et al. (2021) [41] and Dai et al. (2022) [27]. The main damage area of this earthquake is the Jiuzhaigou scenic area, which is also the main area involved in the intensity IX zone in this study. Therefore, the changes in mobile signaling data can reflect the damage degree of the earthquake.

### 4.3. Determination of the Seismic Influence Field by Mobile Phone Signaling

Our results showed a significant correlation between the decommission rate of mobile signaling and the intensity, where the higher the intensity level, the greater the decommission rate of mobile signaling. This mechanism suggests that mobile signaling data can be helpful in determining the seismic influence field. Xia et al. (2019) [40] developed an intensity assessment model based on mobile signaling data from eight earthquakes. In this study, we built four intensity assessment models using the decommission rate with respect to the epicentral distance. In the four models, the epicentral distance and intensity had a negative correlation, while the decommission rate and intensity had a positive correlation. These results are consistent with the relationship between earthquake intensity and these two factors in practice. In terms of model fit, the model built based on Gid had the best model fit, while the model built based on WiFi had the highest accuracy. Of course, since the model developed in this study is only for the Jiuzhaigou earthquake, it is not universally applicable, and there is still a need for further improvement of the model in terms of application to more areas in the future. Therefore, the interpolation analysis using mobile phone signaling data provides a reference for the rapid delineation of the post-earthquake impact field and the emergency response.

### 4.4. Delineating the Seismic Influence Field by Mobile Signaling

The delineation of the seismic influence field is the key to identifying meizoseismal areas for rapid emergency response. The interpolation analysis was carried out based on the decommission rate at 5 min post-earthquake. The result showed that all four signaling types could delineate the impact field to some extent, with the best result being that of the Loginmac. The interpolation results of the Loginmac could determine the meizoseismal areas, and the meizoseismal areas coincided with the published high-intensity zone. The extent and direction of the meizoseismal areas were influenced by the distribution of secondary hazard potential sites and fatalities. However, the direction of the impact field did not coincide with the published intensity map, mainly because the published seismic influence field delineation was influenced by tectonic and geological factors, which were in the same direction of extension as the fracture zone. The interpolation results were mainly influenced by the actual population distribution, while the extension direction was influenced by the potential sites of geological hazards.

### 4.5. Limitations and Challenges

There are inevitably some limitations in data collection and data accuracy because mobile phone signals can be affected by many external factors, such as terrain and communication signal strength. The study area is located in the transition zone from the Qinghai-Tibet Plateau to the Sichuan Basin and features a complex geological background (mainly valley terrain with dense vegetation). Mobile phone signals in some areas may not be accurately captured. For example, in the Jiuzhaigou earthquake-hit area, many tourists were trapped in the No. 122 Forest Farm of Jiuzhaigou County. The mobile phone signals generated by these tourists were thus not captured, and it was difficult to analyze the situation using mobile signaling.

Our results show that changes in mobile phone signaling data can provide a helpful reference for the rapid determination of the seismic influence field. Because the changes in mobile phone signaling data reflect real-time changes in the population distribution, analysis based on mobile phone signaling data can more accurately reflect the extent of the actual meizoseismal areas. Additionally, the data used in this study were 5-bit GeoHash precision, which might be too coarse to assess the seismic influence field. In the future, we will try to use much higher accuracy mobile signaling data to model the seismic influence field. By comparing the assessment results based on data with different precisions, the effects of data precision on the determination of the seismic influence field can be evaluated to further improve the accuracy and applicability of the results.

## 5. Conclusions

This paper analyzed the changes in four types of mobile signaling data between the day before and the day of the Jiuzhaigou earthquake and their relationship with the seismic influence field. The results showed that daily changes in the mobile phone signaling data reflected people’s daily work and rest patterns. The number of devices associated with four mobile signaling types changed abruptly in response to a seismic event. Among the four signaling types, WiFi and Loginmac showed significant recovery after the earthquake. The variation in the number of devices associated with the signaling types varied in different intensity areas. The higher the intensity was, the greater the change and the longer it took to return to a normal state. The magnitude of the change in the Loginmac and WiFi data showed significant changes in all four intensity zones, while Gid and Station data varied significantly only in the high-intensity zones.

Based on the decommission rate of mobile signaling data and the epicentral distance, this study established a rapid assessment model of earthquake intensity. Although there are some data limitations in this study, it provides a useful reference for the rapid estimate of the seismic influence field after an earthquake in China. The intensity assessment model attempted in this study is based only on the Jiuzhaigou Mw 6.5 magnitude earthquake. The reliability and validity of the model, when applied to other regional earthquakes, need to be further confirmed by a number of empirical studies. However, on the basis of obtaining the appropriate data, the model can be applied in other regions. In addition, an interpolation analysis was carried out using kriging interpolation based on mobile signaling data. The interpolation results coincided with the existing intensity maps and were more indicative of the distribution of fatalities and potential secondary hazards. Therefore, the analysis based on mobile phone signaling data can more accurately reflect the extent of the actual meizoseismal areas.

Mobile signaling data have the advantages of spatial continuity, timely accessibility and large data volume. This technique provides a novel methodological idea for the rapid determination of seismic influence fields. Moreover, mobile signaling data contain rich location information. Further mining after anonymization will play an essential role in post-disaster emergency response and rescue.

## Figures and Tables

**Table 1:** Total number and decommission rates of mobile signaling.

Indicators	Gid	Station	WiFi	Loginmac
Day before the earthquake	93,482	65,056	597,533	106,275
Day of the earthquake	67,292	48,405	321,410	46,299
Decommission rate	28%	25%	56%	46%

Note: Day before the earthquake refers to 7 August 20:00–8 August 0:19 time period; day of the earthquake refers to 8 August 20:00–9 August 0:19 time period.

**Table 2:** Number of GeoHash grids of the four types of signaling in different intensity areas.

Indicators	IX	VIII	VII	VI
Gid	4	13	36	59
Station	4	13	31	33
WiFi	4	10	35	53
Loginmac	4	10	26	51

**Table 3:** Four types of signaling sudden changes.

Signaling	Abrupt Change Point	Change Trends	Duration of Sudden Change
Gid	21:20	sudden increase	4 min
	21:21	sudden reduction	
Station	21:20	sudden increase	4 min
	21:21	sudden reduction	
Loginmac	21:20	sudden reduction	5 min
WiFi	21:21	sudden reduction	3 min

**Table 4:** Number of devices associated with the four indicators in different intensity zones.

Signaling Data		IX-Intensity Zone	VIII-Intensity Zone	VII-Intensity Zone	VI-Intensity Zone
Gid	Day before the earthquake	19,242	22,346	7688	44,206
	Day of the earthquake	9389	13,177	5504	39,222
	Decommission rate	51.20%	41.03%	28.40%	11.27%
Station	Day before the earthquake	11,923	14,290	5509	33,334
	Day of the earthquake	6128	8799	4301	29,177
	Decommission rate	48.60%	38.42%	21.92%	12.47%
Loginmac	Day before the earthquake	16,204	17,564	7953	64,554
	Day of the earthquake	2445	4151	1704	37,999
	Decommission rate	84.91%	76.36%	78.57%	41.13%
WiFi	Day before the earthquake	139,896	198,243	23,431	235,963
	Day of the earthquake	44,032	77,016	9335	191,027
	Decommission rate	68.52%	61.15%	60.15%	19.04%

**Table 5:** Correlations between the intensity of mobile signaling and other variables.

Variables	*D* *_gi_*	*R* *_gi_*
*I* *_g_*_1_	−0.900 **	0.244 *
*I* *_g_*_2_	−0.886 **	0.278 *
*I* *_g_*_3_	−0.894 **	0.474 **
*I* *_g_*_4_	−0.880 **	0.385 **

Where ** indicates a significance level of 0.01 (two-tailed) and * indicates a significance level of 0.05 (two-tailed).

**Table 6:** Results of regression analysis with different mobile phone signaling types.

Models	Adjusted R^2^	Sig.	Accuracy
(1) *I*_1_ = 8.662 − 0.062 × *D*_1_ + 0.432 × *R*_1_	0.822	0.000	84%
(2) *I*_2_ = 8.306 − 0.054 × *D*_2_ + 0.45 × *R*_2_	0.786	0.000	82%
(3) *I*_3_ = 8.229 − 0.052 × *D*_3_ + 0.623 × *R*_3_	0.771	0.000	86%
(4) *I*_4_ = 8.233 – 0.050 × *D*_4_ + 0.357 × *R*_4_	0.749	0.000	82%

## Data Availability

The data used in this study are available from the corresponding author upon reasonable request.
